# Combe’s Lectures on Phrenology

**Published:** 1840

**Authors:** 


### COMBE'S PHRENOLOGICAL LECTURES.

The celebrated GEORGE COMBE, Esq., of Edinburg, who came to the United States, twelve or eighteen months ago, to lecture on phrenology, after delivering several courses, which were well attended, in the cities of the east, lately made a flying visit in the west. His stay in Pittsburgh, Cincinnati, Louisville and Lexington, respectively, was exceedingly brief, and he did not deliver a single lecture in either place. We understand that he is about to return to Scotland. He is said to have made many converts to Phrenology in Philadelphia, New York and Boston, where indeed, the number previously existing was considerable. D.

